# Case series: “Double arch” changes caused by capsule contraction syndrome after cataract surgery in highly myopic eyes

**DOI:** 10.1186/s12886-021-02113-z

**Published:** 2021-10-18

**Authors:** Wei Wang, Dejian Xu, Xin Liu, Wen Xu

**Affiliations:** 1 Eye Center, Second Affiliated Hospital of School of Medicine, Zhejiang University, No. 88 Jiefang Road, Hangzhou, China; 2 Department of Ophthalmology, Taizhou Municipal Hospital, Taizhou, China

**Keywords:** Double arch, Capsule contraction syndrome, Optic deformation, Highly myopic

## Abstract

**Background:**

Capsule contraction syndrome (CCS) after cataract surgery causes intraocular lens (IOL) haptic flexion and IOL optic displacement in most former reports. However, there are few reports on CCS-induced deformation of the IOL optic. We report two cases of CCS after cataract surgery in highly myopic eyes and describe a previously unreported “double arch” complication.

**Case presentation:**

Two patients with history of high myopia had cataract surgery with hydrophilic acrylic plate haptic IOLs implanted in their eyes. CCS with arch shape deformation of the pupil as well as the optic of the IOL were noticed in both cases after three months, which induced refractive changes and corrected distance visual acuity (CDVA) deterioration. Visual acuity of the patients was restored by replacing the IOL from the capsular bag to the ciliary sulcus and the following neodymium: YAG (Nd:YAG) laser capsulotomy. We propose that such “double arch” change brought by CCS is related to the plate-haptic design of the IOL and the incomplete overlap between the capsular opening and the IOL optic.

**Conclusions:**

We recommend careful IOL selection and proper capsulorhexis in patients with high myopia or with other risk factors of CCS. Early diagnosis and timely treatment of CCS are critical to prevent visual symptoms and further ocular complications.

## Background

Capsule contraction syndrome (CCS) is one of the known complications after cataract surgery. It usually develops within the first 3 months after operation [1]. The anterior capsule opacity(ACO) and shrinkage may decrease visual function [2]. The development of CCS can lead to IOL decentration [3], IOL dislocation [4], and even ciliary body detachment [5]. Previous studies showed that CCS was more prevalent among patients with some preexisting ocular or systemic disorders, such as high myopia [6], retinitis pigmentosa [7] and diabetes mellitus [8]. The material of IOLs have also been reported to be associated with CCS development [9]. It had been proved that silicone IOLs lead to a greater degree of capsule contraction than acrylic IOLs, and that the incidence of CCS was significantly higher when implanting hydrophilic IOLs compared to hydrophobic IOLs. However, the influence of the optic and haptic design on CCS is controversial [1].

The CT Asphina 509 M IOL(Carl Zeiss Meditec AG, Jena, Germany) is a one-piece hydrophilic acrylic IOL with an optic diameter of 6 mm and an overall length of 11 mm. It has a 360-degree square edge and two plate-shaped haptics at a 180° interval around the optic. It is designed to fit into a 1.8 mm corneal incision [10]. Theoretically, the plate-haptic IOL would center more accurately in the capsular bag. However, up to now, few studies report its long-term clinical stability. Here, we report two cases of capsule contraction syndrome in highly myopic eyes implanted with CT Asphina 509 M IOL that displayed a previously unreported “double arch” deformation.

## Case presentation

### Case 1

**Fig. 1:**
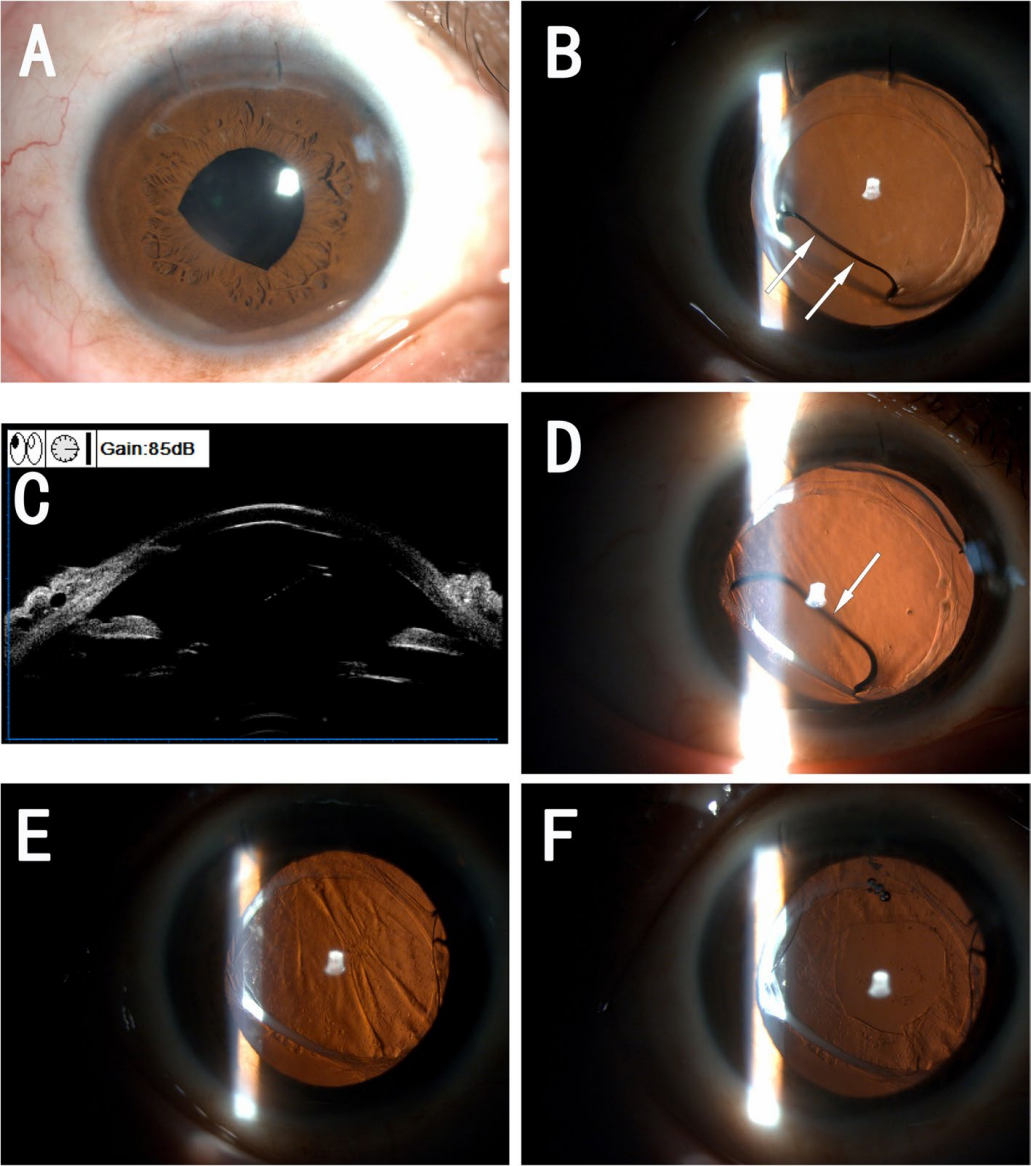
The slit-lamp examination showed arch shape change and pupil capture of the IOL (**A**). Mydriatic examination showed anterior capsule opacity and fibrosis (**B**), with arch shape change of the IOL optic (**B**, white arrow). Ultrasound biomicroscopy (UBM) showed the optic bent into the anterior chamber, close to the cornea endothelium (**C**). After Nd:YAG laser anterior capsulotomy, IOL deformation was not improved, while local corneal edema appeared (**D**, white arrow). The slit-lamp examination showed posterior capsule relaxation and opacity at one week after IOL adjustment surgery (**E**). The IOL remained stable after surgery and Nd:YAG posterior capsulotomy (**G**)

A 41-year-old female patient with a history of high myopia and refractive surgery of anterior chamber IOL(AC-IOL) implantation in phakic eyes was diagnosed with a nuclear cataract in her right eye. The axial length of the right eye was 31.44 mm. Uneventful AC-IOL explantation and phacoemulsification cataract extraction was performed (W.X.), and a CT Asphina 509 M IOL with a power of + 2 D was implanted. One week after the surgery, the corrected distance visual acuity(CDVA) was 20/50, with a refraction of -2.00/-2.00*85. Neither visual discomfort nor structural abnormality of the operated eye were detected in the postoperative 1 week and 1 month. However, the patient complained of blurred vision at 4 months postoperatively. Her CDVA of the right eye decreased to 20/200, with a refraction of -1.50/-2.50*77. A slit-lamp examination revealed an arch-shape change of the pupil, along with a pupil capture of the IOL (Fig. 1A). After pupil dilation, fibrosis and opacity of the anterior capsule ring with obvious IOL tilt and deformation were noted (Fig. 1B). The IOL optic was squeezed into arch-shape and the inferior part of it curled out of the capsular bag. Ultrasound biomicroscopy(UBM)(SW-3200, Suoer, Tianjin, China) examination showed that the IOL optic bent into the anterior chamber, approaching the cornea endothelium (Fig. 1C). We then performed a neodymium:YAG(Nd:YAG) laser anterior capsulotomy with a radial relaxing incision to enlarge the anterior capsule opening, hoping that the IOL can be expanded and reset into the capsule bag. However, the force of the shrunk anterior capsule opening is so strong that the IOL extruded even further into the anterior chamber. Local cornea edema appeared 2 days after laser treatment (Fig. 1D), suggesting that the IOL may have contacted the corneal endothelium. Consequently, we decided to adjust the IOL position by surgery. After a 2.0 mm clear cornea incision, an ophthalmic viscosurgical device(OVD) was injected, then a venous scissor was used to cut the anterior capsule radially to loosen the capsule contraction. However, the bent structure of the IOL and the synechia between the plate haptic and capsule prevented us from separating and rotating the IOL. Finally, the haptics were pulled out from the capsular bag by the viscoelastic needle and placed in the sulcus. Then OVD was removed by irrigation/aspiration(I/A), and the incision was hydrated with a balanced salt solution. One week after the adjustment, however, her blurred vision persisted, with a CDVA of 20/100. A careful slit-lamp examination revealed posterior capsule folds and the opacity of this patient. (Fig. 1E) Successful Nd:YAG laser posterior capsulotomy was performed. One week later, her CDVA reached 20/50 with a refraction of -3.00/-2.75*98. The patient was satisfied with her improvement in vision. The IOL remained stable (Fig. 1F) on the follow-up visit of 3 months after the adjustment surgery.

### Case 2

**Fig. 2:**
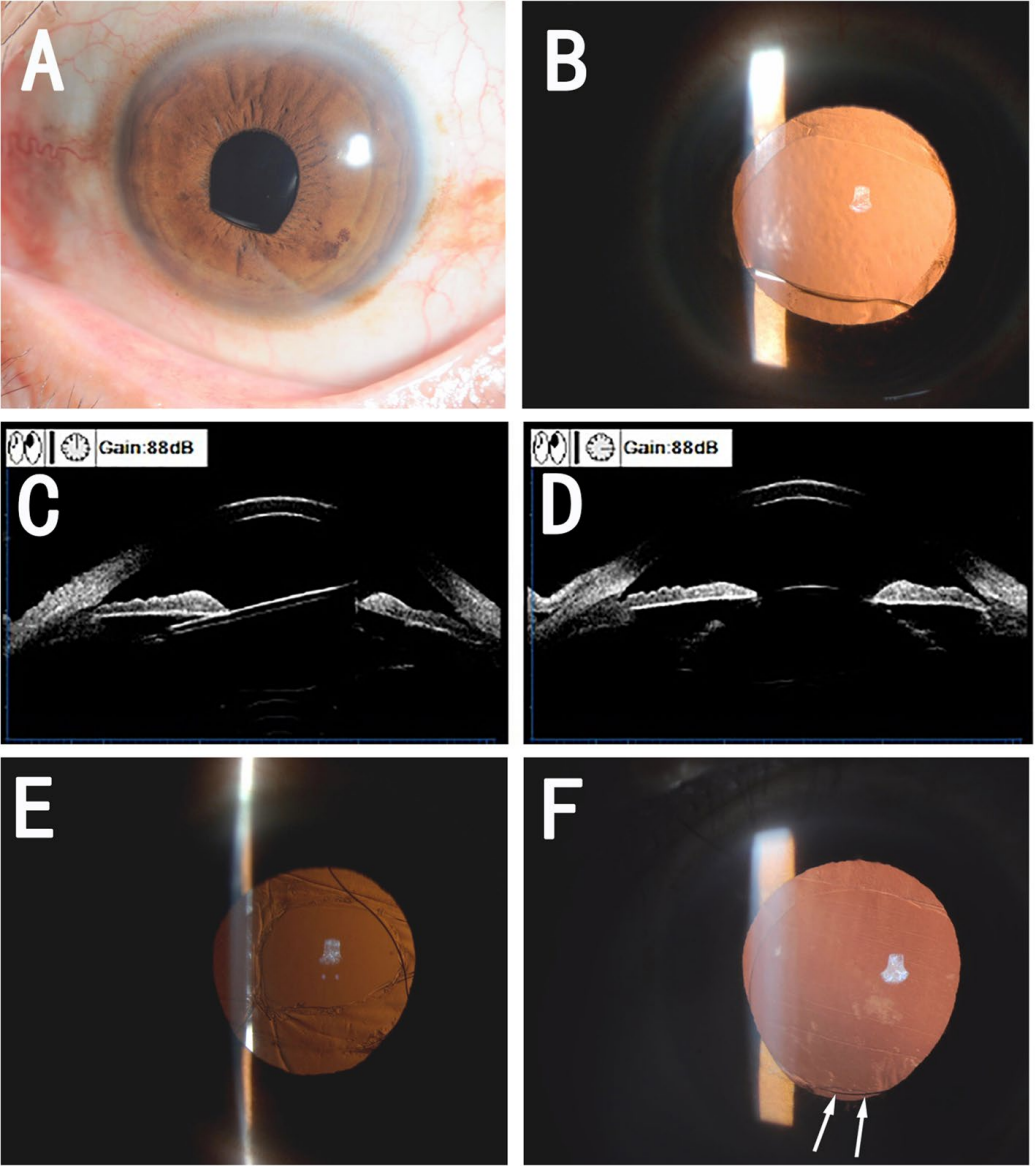
The slit-lamp examination showed arch shape change and pupil capture of the IOL (**A**). A mydriatic examination showed anterior capsule opacity and fibrosis (**B**), with IOL optic deformation. UBM showed IOL tilted along the vertical axis (**C**) while bent along the horizontal axis (**D**). The IOL remained stable after surgery and Nd:YAG posterior capsulotomy (**E**). A mydriatic examination at one week after cataract surgery showed an incomplete overlap of the inferior IOL optic (**F**, white arrow)

A 61-year-old male patient with a history of high myopia had uneventful micro-incision cataract surgery(MICS) with phacoemulsification (W.X) in his left eye. His preoperative axial length was 32.8 mm. During surgery, a continuous curvilinear capsulorhexis of approximately 5.5 mm in diameter was performed, and a CT Asphina 509 M IOL with the power of + 3 D was implanted into the capsular bag. The immediate postoperative condition was uneventful, and the CDVA after 1 month was 20/25 with a refraction of -3.25/-0.75*27. However, his CDVA decreased to 20/50 with a refraction of -2.25/-1.50*155 at 3 months postoperatively. A slit-lamp examination revealed an arch-shape change of the pupil, similar to case 1 (Fig. 2A). After pupil dilation, an anterior capsule contraction and an arch-shape change of the IOL were noted (Fig. 2B). A UBM examination showed that IOL was bent outside the capsule (Fig. 2C,D). Based on our experience from the first patient, we performed adjustment surgery to place the IOL into the ciliary sulcus. One week after the surgery, the CDVA of this patient was 20/100, and the slit-lamp examination detected posterior capsule relaxation and opacity. A Nd:YAG laser posterior capsulotomy was performed (Fig. 2E). One week later, his CDVA reached 20/25 with a refraction of -3.75/-0.50*20. The patient was satisfied with his improvement in vision. We reviewed the patient’s mydriatic photograph at 1 week after cataract surgery and noticed that the inferior edge of the IOL optic was not overlapped by the anterior capsule opening (Fig. 2F).

## Discussion

We described a unique CCS-induced “double arch” change after cataract surgery. It features in the arch shape deformation of both the pupil and the IOL optic, leading to ametropia and poor visual acuity. We believe that the occurrence of CCS in both cases are related to high myopia and the IOL feature.

High myopia is recognized as one of the most important risk factors of CCS. Zhu et al. [11] identified elevated aqueous humor TGF-b2 levels in aqueous humor as well as upregulated TGF-bRII expression in lens cells among highly myopic cataract patients, which might contribute to the pathogenesis of anterior capsule contraction through activating the transdifferentiation of lens cells into myofibroblasts. On the other hand, the weak zonular structure in highly myopic eyes exacerbated the imbalance between the centripetal force of the anterior capsule opening margin (fibrotic change) and the centrifugal force of the capsular zonules [12], making it vulnerable to CCS risk after cataract surgery.

In addition to the history of high myopia, we propose that the IOL we used in the cases could be a predisposing factor for capsule contraction syndrome. The inherent flexibility of hydrophilic materials makes the CT Asphina 509 M IOL more susceptible to deformation when capsule contraction syndrome happens. Designed to be compressed easily to fit through microincision, such IOLs also has a soft and thin surface. In addition, IOL with lower power that are suitable for highly myopic eyes present flatter optic surfaces. All the above features of the IOL applied in our cases could lead to weak resistance towards capsule contraction. In most of the former reports, CCS causes IOL haptic flexion and IOL optic displacement, thereby leading to visual discomfort [13]. So far, only one case of CCS-induced IOL optic bent has been reported [14], which involved a CT Asphina 509 M IOL implanted in a high myopia eye. In that case, the IOL optic bent backward due to capsule contraction syndrome, leading to a hyperopic shift and obvious internal astigmatism. The authors pointed that the plate haptic design implies a large surface in contact with the capsular bag, thus triggering capsular reaction. Choi et al. compared three hydrophilic acrylic IOLs and found that contraction of the anterior capsule opening was much smaller with the four-loop haptic IOL than with the two-loop haptic and two-plate haptic IOLs [10]. On the other hand, unlike loop haptics, plate haptics could not adapt to the contracted capsule easily. Therefore, the surface of the optic is more likely to tilt or bend when the centripetal force is transmitted from the plate haptic rather than from the loop haptic. Thus for patients with high risk of CCS, we recommend implanting hydrophobic IOLs. Compared with plate-haptic IOLs, we suggest that IOLs with loop-haptics are better choices for these patients.

**Fig. 3:**
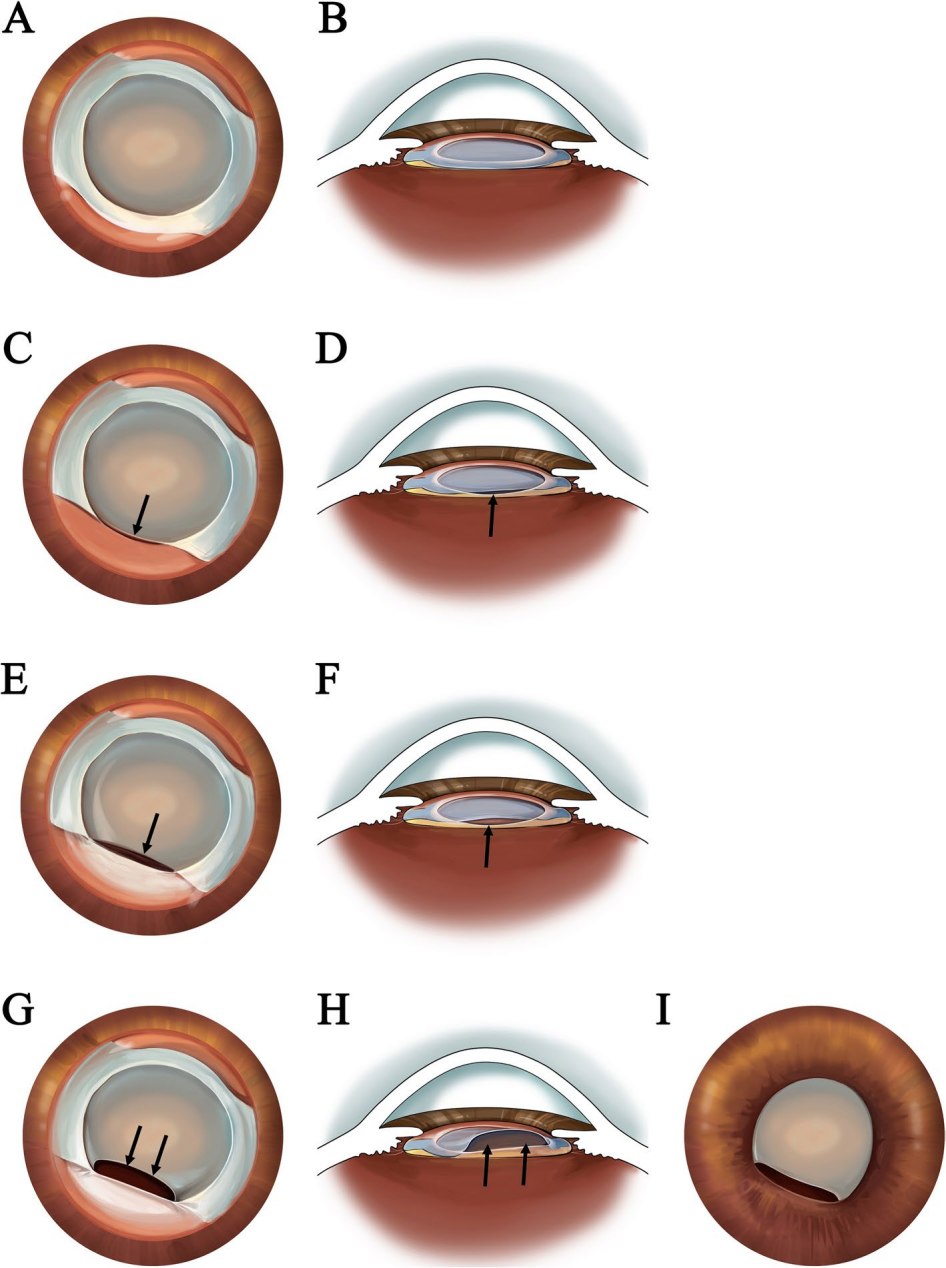
The hypothesis of double arch development. Ideal IOL position: the well-centered and optic edge is completely overlapped by the anterior capsular opening (**A**, **B**). Stage 1: Part of the optic edge was not overlapped and escaped from the capsular bag (**C**, **D**, black arrows). Stage 2: With the fibrosis and contraction of the anterior capsule, the escaped IOL optics were squeezed and moved forward (**E**, **F**, black arrows). Stage 3: As the capsule opening shrinks further, the IOL optic deforms and bends beyond the pupil plane (**G**, **H**, black arrows), and thus appear the double arch changes (**I**)

Contrary to those previously reported cases of CCS-induced IOL haptic deformation, the anterior capsule opening had not shrunk to tiny sizes before the IOL optics deformed in our cases. This suggests that the occurrence of “double arch” changes may have a special mechanism. Based on the postoperative photos and the UBM inspection results of the patients, we suppose that the double arch deformation may be related to the plate-haptic design of the IOL and the incomplete overlap between the anterior capsular opening and the IOL optic. We speculate the following possible steps of the double arch development:

- Stage 1: The IOL optic is not completely overlapped with the capsular bag (Fig. 3C, D).
- Stage 2: With the contraction of the anterior capsule opening, pressure is applied to the edge of the IOL optic, and centripetal force is generated by CCS transmitted from the plate haptic to the optic, which causes the bent and arch shape change of the relatively thin and soft optic (Fig. 3E,F).
- Stage 3: When the optic arches beyond the pupil plane (Fig. 3G,H), pupil capture of the IOL occurs, which in turn causes the arch shape deformation of the pupil (Fig. 3I).

We propose that the incomplete overlap of the IOL optic is one of the main reasons for the double arch changes. Deviated or large capsulorhexis, as well as intraocular pressure fluctuations during the watertight process or at the early postoperative stage, may cause part of the IOL optic edge to escape from the capsule bag. Therefore, we emphasize that for patients who are prone to CCS, a well-centered and appropriately sized capsulorhexis is very important, especially when implanting with plate-haptic IOL. In addition, a capsular tension ring (CTR) could be implated in these patients to prevent severe anterior capsule contraction and IOL decentration or tilt [15]. During the watertight process, excessive water flow should be avoided, and the incision should be well hydrated to avoid postoperative leakage.

Treatment of CCS varies depending on the degree of visual disturbance, the stability of capsule and IOL, and the co-existence of other complications [1]. Nd: YAG laser anterior capsulotomy is suggested as the first-line treatment for capsule contraction syndrome. In the first patient, however, the severe deformation of the optical part and the pressure of the capsular opening on the optical part failed to flatten the IOL after laser anterior capsulotomy. Therefore, we suggest that for stage 1 of patients with a high risk of CCS, mydriatic examination and close follow-up is necessary, while attention should be paid to the potential occurrence of the double arch change. CCS with optics deformation at stage 2, on the other hand, should be treated with laser anterior capsulotomy to avoid further complications. When CCS reaches stage 3 to cause double arch change, where the IOL haptics had firmly adhered to the capsular bag and the IOL optic surface had severely deformed, surgical IOL adjustment is required.

## Conclusion

According to our cases, we suggest that IOL with hydrophilic texture, softer surface, flatter optics, and plate-haptic design should be implanted with caution in patients with a high risk of CCS, such as those with high myopia. Surgeons need to make a properly sized and well-centered capsulorhexis during surgery and ensure that the IOL optic is completely overlapped with the capsular opening at the end of the surgery. A mydriatic examination of the anterior capsule and IOL position is critical at the early stage after cataract surgery. Once the tendency of CCS is noted, close follow-up and timely treatment should be carried out to avoid serious complications.

## Data Availability

All data generated and analyzed during this study are included in this article.

## Abbreviations

CCS: Capsule contraction syndrome
IOL: Introcular lens
ACO: Anterior capsule opacity
AC-IOL: Anterior chamber IOL
CDVA: Corrected distance visual acuity
UBM: Ultrasound biomicroscopy
NdYAG: Neodymium:YAG
OVD: Ophthalmic viscosurgical device
I/A: Irrigation/aspiration
MICS: Microincision cataract surgery
CTR: Capsular tension ring
